# Co-inheritance of recombined chromatids maintains heterozygosity in a parthenogenetic ant

**DOI:** 10.1038/s41559-024-02455-z

**Published:** 2024-07-16

**Authors:** Kip D. Lacy, Taylor Hart, Daniel J. C. Kronauer

**Affiliations:** 1 Laboratory of Social Evolution and Behavior, The Rockefeller University, New York, NY USA; 2 Howard Hughes Medical Institute, New York, NY USA

**Keywords:** Evolutionary genetics, Evolutionary biology

## Abstract

According to Mendel’s second law, chromosomes segregate randomly in meiosis. Non-random segregation is primarily known for cases of selfish meiotic drive in females, in which particular alleles bias their own transmission into the oocyte. Here we report a rare example of unselfish meiotic drive for crossover inheritance in the clonal raider ant, *Ooceraea biroi*, in which both alleles are co-inherited at all loci across the entire genome. This species produces diploid offspring parthenogenetically via fusion of two haploid nuclei from the same meiosis. This process should cause rapid genotypic degeneration due to loss of heterozygosity, which results if crossover recombination is followed by random (Mendelian) segregation of chromosomes. However, by comparing whole genomes of mothers and daughters, we show that loss of heterozygosity is exceedingly rare, raising the possibility that crossovers are infrequent or absent in *O. biroi* meiosis. Using a combination of cytology and whole-genome sequencing, we show that crossover recombination is, in fact, common but that loss of heterozygosity is avoided because crossover products are faithfully co-inherited. This results from a programmed violation of Mendel’s law of segregation, such that crossover products segregate together rather than randomly. This discovery highlights an extreme example of cellular ‘memory’ of crossovers, which could be a common yet cryptic feature of chromosomal segregation.

## Abstract (web summary)

This study reports non-random segregation of chromosomes during meiosis in the clonal raider ant, *Ooceraea biroi*, but no loss of heterozygosity because crossover products are faithfully co-inherited.

## Main

Meiosis and sex are ancient, predating the last common ancestor of eukaryotes^1^. Nevertheless, animal lineages that reproduce via parthenogenesis (development from eggs without fertilization by sperm) evolve sporadically via mutations that perturb meiosis. The underlying alterations to the meiotic programme are rarely understood but might provide insights into the evolution of asexuality and the fundamental mechanisms of meiosis.

Parthenogenetic lineages face two major challenges: (1) they must restore euploidy without sexual fertilization and (2) they must produce offspring with viable genotypes. This often requires maintenance of heterozygosity to avoid recessive lethality, preserve genetic diversity and transmit genotypes compatible with female development^2–6^. The many known parthenogenetic lineages use a variety of cytological mechanisms for ploidy restoration^7,8^ but how they maintain heterozygosity is usually unknown.

**Fig. 1:**
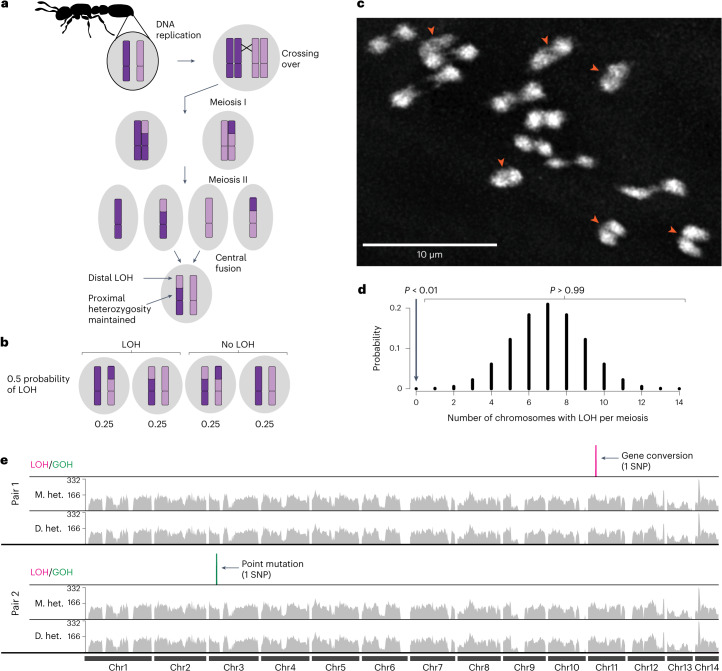
Loss of heterozygosity should be frequent but is rare in *O. biroi.* **a**, Schematic of *O. biroi* parthenogenesis, depicting the fusion of central haploid pronuclei to form the diploid zygote and the expected loss of heterozygosity distal to crossovers. Dark and light purple colouring on chromosomes indicates allelic identity. **b**, Depiction of all possible genomic compositions of diploid zygotes following segregation of a crossover and central fusion, along with their expected probabilities. **c**, Maximum projection of DAPI-stained *O. biroi* chromosomes at metaphase I, with pairs of homologues connected by chiasmata. Red arrowheads indicate clearly visible chiasmata. This was repeated for five metaphase I oocytes with consistent results. **d**, Probability mass function of a binomial model describing how many chromosomes are expected to incur a loss of heterozygosity per meiosis, with one crossover on each of the 14 homologous chromosomes in *O. biroi*. Shown above are the probabilities that loss of heterozygosity occurs on no chromosomes (*P* = 0.00006) or on one or more chromosomes (*P* = 0.99994). **e**, Karyoplot depicting all SNPs that underwent loss of heterozygosity (magenta) or gain of heterozygosity (green) among the sequenced genomes from two mother–daughter pairs. The expected segmental losses of heterozygosity were notably absent. For each individual, grey histograms show the number of sites heterozygous in 250 kb windows. LOH, loss of heterozygosity; GOH, gain of heterozygosity; M. het., mother’s heterozygosity; D. het., daughter’s heterozygosity. Source data

The clonal raider ant, *Ooceraea biroi* (formerly *Cerapachys biroi*), has satisfied these dual challenges. In this species, diploid offspring are formed via the fusion of two non-sister haploid pronuclei, each originating from different meiosis II divisions within the same meiotic cycle (Fig. 1a)^9^. Genetically, this means that chromatids from two different homologous chromosomes (rather than two identical sister chromatids) are inherited, except in cases of recombination. This mode of parthenogenesis, known as ‘automixis with central fusion’, solves the problem of ploidy restoration but creates a problem for heterozygosity maintenance distal to crossovers (Fig. 1a).

Crossovers provide the physical tension that ensures accurate chromosome segregation in meiosis. Accordingly, most species have at least one crossover per chromosome (crossover assurance). Following a crossover, chromosomes are thought to segregate randomly (Mendel’s second law). In central fusion parthenogenesis, there are four possible inheritance outcomes following a crossover and random segregation means that they are expected to occur with equal probability (Fig. 1b). Two of these outcomes lead to segmental loss of heterozygosity due to the inheritance of one crossover product and one non-crossover product. Thus, we expect half of all crossovers to produce a segmental loss of heterozygosity. If crossovers occur on each of the 14 homologous chromosomes in *O. biroi*^10,11^, then loss of heterozygosity should occur commonly.

In contrast to the expectation that heterozygosity should be lost rapidly, genetic studies suggest that little, if any, heterozygosity is lost each generation^9,12^. How heterozygosity is maintained has remained a mystery. One possibility is that *O. biroi* meiosis lacks crossovers. Crossovers are required in canonical meiosis^13^ but are absent from male or female meiosis in several insect taxa (sex-limited achiasmy)^14^. On the other hand, if crossovers occur normally, non-Mendelian inheritance, that is, non-random segregation of chromosomes during meiosis^15–17^, could ensure heterozygosity maintenance. Alternatively, losses of heterozygosity might be inherited but go unnoticed because they are lethal^18,19^. Here we uncover the mechanism of heterozygosity maintenance in *O. biroi* using a combination of cytology, genome sequencing and modelling.

## Results

### Loss of heterozygosity should be common but it is rare

**Extended Data Fig. 1:**
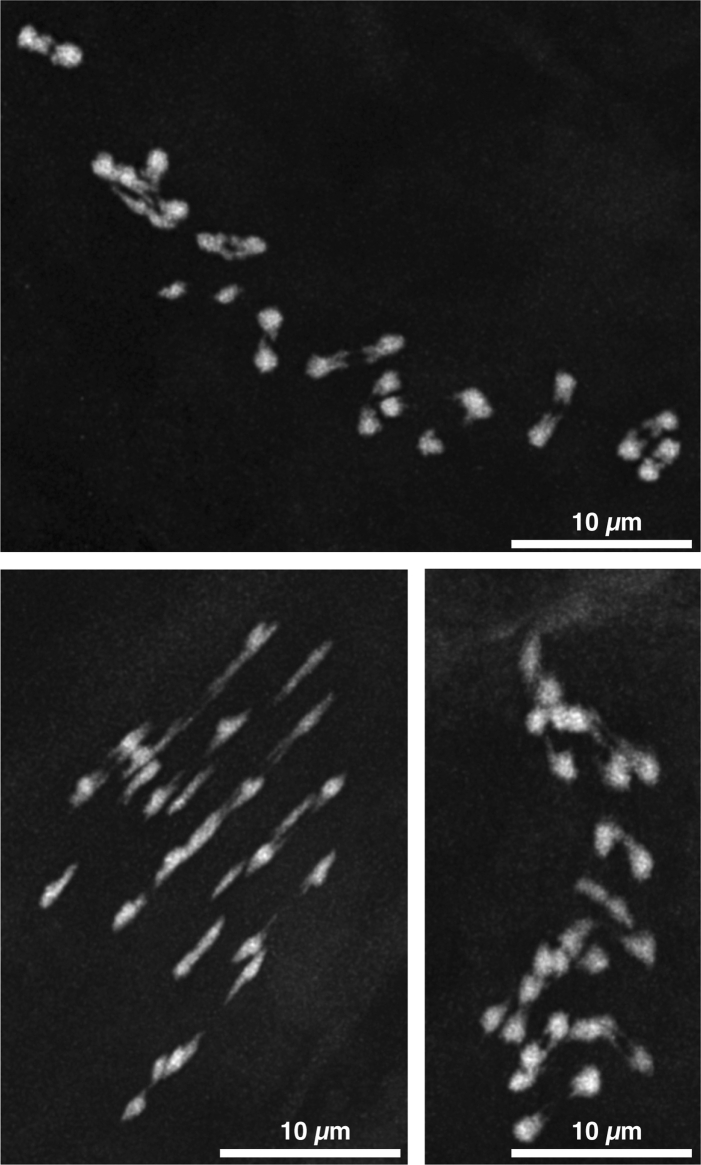
Additional metaphase I spreads reveal that chiasmata connect paired homologs. Maximum projections of DAPI-stained *O. biroi* chromosomes at metaphase I reveal DAPI connections between paired homologs that indicate chiasmata. Each projection is from a different oocyte produced by a different ant. Additional replicate oocytes are in Fig. 1c and Supplementary Video 1.

To investigate whether *O. biroi* meiosis lacks crossovers, we performed chromosome squashes during metaphase I. These cytological preparations can make physical crossover structures (chiasmata) visible as DNA connections between paired homologous chromosomes^20,21^. Such DNA connections are absent in species that lack meiotic crossovers^22^. We found that paired homologous chromosomes were linked by positive DAPI staining, indicative of chiasmata (Fig. 1c, Extended Data Fig. 1 and Supplementary Video 1). Although not definitive, this suggests that crossovers occur normally in *O. biroi* meiosis, which means losses of heterozygosity should be frequent.

How frequent should losses of heterozygosity be, exactly? To answer this question, we used the binomial distribution, which is a good fit because (1) assuming Mendelian segregation, each crossover has a fixed probability of 0.5 of leading to segmental loss of heterozygosity and (2) homologous chromosomes comprise a set of independent trials. In this way, we obtained the probability distribution of the number of chromosomes that undergo at least one loss of heterozygosity each meiosis (Fig. 1d). This distribution showed that loss of heterozygosity should typically occur on several chromosomes, with a >99% chance of occurring on at least one chromosome.

**Extended Data Fig. 2:**
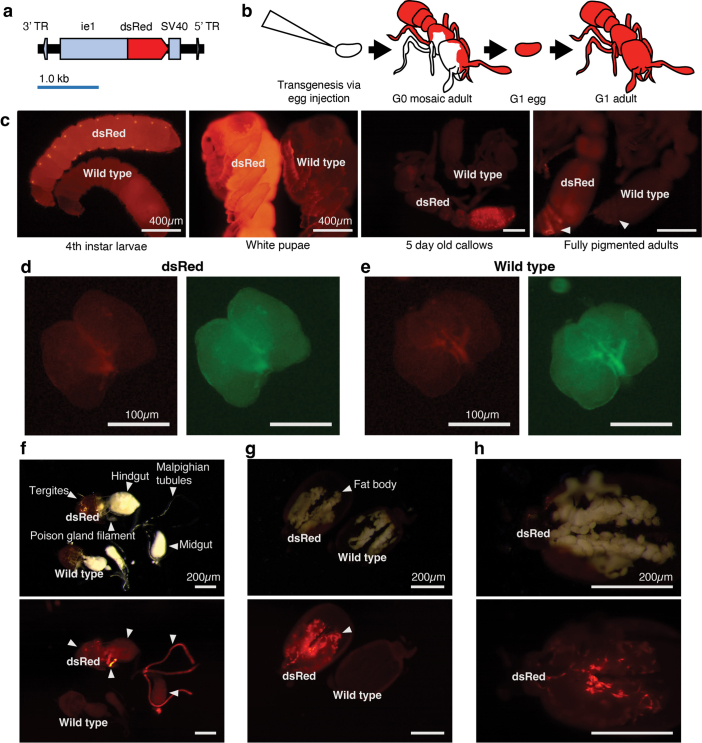
Generating the transgenic line. **a**, Diagram of the transgene construct pBac-ie1-dsRed. Promoter/enhancer ie1, dsRed coding sequence and SV40 termination sequence were assembled between the 3’ and 5’ Terminal Repeats (TRs) on a piggyBac plasmid backbone. **b**, Schematic for generating transgenic clonal raider ants. Young eggs are collected from wild-type (WT) ants and injected with transposase/vector mix. Some eggs develop into G0s with somatic mosaicism of the integrated transposon. G0s are reared in groups and screened for fluorescence. Eggs are collected from the mixed population of G0s and reared to produce G1s with potentially unique transgene integrations. **c**, Fluorescent phenotypes of transgenic clonal raider ants. In each panel, a transgenic individual from clonal line B is shown next to a WT individual from clonal line B at the same developmental stage, imaged under epifluorescence. Left to right: fourth instar larvae, pupae, five-day-old callows and fully pigmented adults. In the rightmost panel, the arrows indicate the region of brightest fluorescence in dsRed individuals and the corresponding spot in WTs, respectively. Fluorescence in transgenic individuals is visible in live individuals across all life stages, excluding eggs. **d**,**e**, Brains from transgenic (**d**) and WT clonal line B (**e**) ants were dissected and imaged under epifluorescence. Only autofluorescence is visible in brains, revealing that dsRed is not expressed in brains in this transgenic line. Autofluorescence in the green channel is shown for each image to visualize anatomy (right panels in **d**,**e**,). **f**, One transgenic gaster and one WT clonal line B gaster were dissected side by side, both from 5-day-old callows. Dissected tissues are visible in bright field (top) and dsRed fluorescence is visible under epifluorescence in several tissues, including the poison gland filament and Malpighian tubules (bottom). **g**, Fat body tissue attached to the cuticle of the gaster is visible under bright field in transgenic and WT individuals (top), with dsRed detected in structures associated with the fat body (bottom). **h**, Close-up on the transgenic fat body from (**g**). For panels **c**-**h**, all experiments were repeated for five different ants with consistent results.

**Extended Data Fig. 3:**
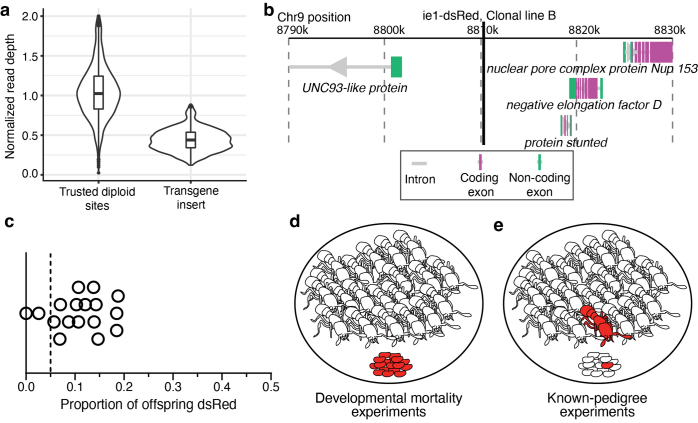
Characterizing and utilizing the transgenic line. **a**, Normalized read depth at a panel of 2,628 trusted diploid heterozygous sites in the *O. biroi* reference genome compared to read depth at all sites in the 2,628 bp transgene insert sequence based on whole genome resequencing of a single transgenic ant. The read depth at the transgene insert was approximately 0.5x the read depth at heterozygous SNPs in the *O. biroi* reference genome, revealing that a single insertion occurred. Box plots show median (center line), interquartile range (IQR) (box limits) and 1.5 x IQR (whiskers). Data points that fall outside 1.5 x IQR are shown individually. Violin plots show the kernel probability density, meaning that the proportion of the data located at a y-axis value is represented by the width of the outlined area. **b**, Genomic context of the mapped insertion site for the transgene on the ninth chromosomal scaffold of the *O. biroi* reference genome. The insertion did not interrupt any annotated genes. **c**, The transgenic line does not have a fitness deficit. Replicate colonies with one transgenic individual and 19 wildtype individuals were allowed to cycle and the proportion of transgenic offspring were counted. The vertical dotted line indicates the expected proportion of transgenic offspring. **d**, **e**, Cartoons of the experimental setups for developmental mortality experiments **d** and known-pedigree-producing colonies **e**. **d**, For developmental mortality experiments, replicate colonies contained 46 fluorescently labeled eggs, 40 WT adults and 38 WT pupae (not shown). By counting the number of fluorescent offspring after eggs had developed, we determined mortality rates while avoiding the possibility of underestimation due to additional egg-laying by adults throughout the experiment. **e**, For known-pedigree production, we housed one fluorescently labeled ant with 19 WT ants and allowed colonies to lay eggs and rear their offspring. We sequenced whole genomes of fluorescently labeled pairs of mothers and daughters or pairs of sisters. This experimental setup was also used for the fitness experiments shown in **c**.

Previous studies reported that loss of heterozygosity was rare but relied on sparse genetic markers and compared individuals of unknown pedigree^9,12^. Ideally, loss of heterozygosity would be quantified from whole genomes from animals of known pedigrees. However, *O. biroi* ants only reproduce as part of a colony and all individuals in a colony lay eggs. Because the species is clonal, it is impossible to assign individual offspring to a particular parent in the absence of unique genetic markers. To overcome this challenge, we created a transgenic clonal line with broad expression of the fluorescent marker protein dsRed (Methods; Extended Data Figs. 2 and 3 and Supplementary Table 1). Using this transgenic line, we obtained two mother–daughter pairs, sequenced the whole genome of each individual and compared these genomes to identify changes in heterozygosity (descriptions and metadata for all genomes sequenced in this study are in Supplementary Table 2). No segmental losses of heterozygosity occurred in either mother–daughter pair (Fig. 1e and Extended Data Table 1). Moreover, no segmental losses of heterozygosity occurred since the common ancestor of the mothers of the two pairs, even though these two genomes were separated by at least four but fewer than 24 meioses. The only changes in heterozygosity found between any of these individuals spanned short genomic tracts, including gains of heterozygosity via point mutations and small losses of heterozygosity due to gene conversion, which occurs at the site of both crossover and non-crossover recombination and affects regions typically <1,500 base pairs (bp) in length^23,24^ (Extended Data Table 2). One mother–daughter pair suffered loss of heterozygosity at a single single-nucleotide polymorphism (SNP), probably due to gene conversion; the other pair gained heterozygosity at a single SNP due to a point mutation. Between the two mothers, there were 12 short losses of heterozygosity consistent with gene conversion and two gains of heterozygosity due to point mutations. These data reveal that, paradoxically, segmental loss of heterozygosity is extremely rare. This finding is in accord with previous demonstrations of heterozygosity maintenance in all known *O. biroi* clonal lines under both laboratory and field conditions^9,12,25,26^.

### Crossovers occur every meiosis without losing heterozygosity

**Fig. 2:**
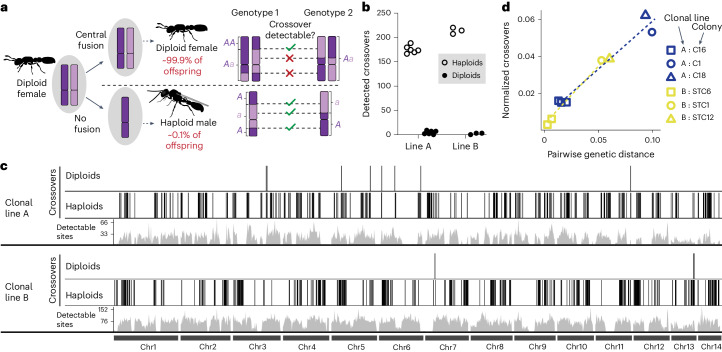
Haploid male genomes indicate that crossover recombination is common. **a**, Haploid males occur rarely in *O. biroi* but can be used to detect crossover recombination. Light and dark purple colouring on chromosomes indicates allelic identity and purple lettering next to chromosomes represents genotypes inferred from whole-genome sequencing. A comparison of example genotypes between two individuals illustrates that, using short-read sequencing, more crossovers are detectable between haploid genomes than between diploid genomes. This is because crossover recombination is directly observed in haploid males and inferred from segmental losses of heterozygosity in diploid females. Therefore, crossovers not followed by loss of heterozygosity cannot be detected among diploid genomes. **b**, The numbers of crossovers detected among all pairwise comparisons of haploid male and diploid female genomes. We sequenced four haploids and four diploids from a single stock of clonal line A, yielding six pairwise comparisons, and three haploids and three diploids from a stock of clonal line B, yielding three pairwise comparisons. **c**, Karyoplot illustrating all detected crossovers (dark vertical bars) and the number of sites ancestrally heterozygous in 200 kb windows (‘Detectable sites’; grey histograms). Closely spaced vertical bars (crossovers) appear as thick black blocks. Many more crossovers are detected among haploids than among diploids. **d**, Jittered scatterplot depicting the correlation of crossover recombination with genetic distance in two *O. biroi* clonal lines. Within each clonal line, each point represents the number of crossovers observed between each male and a randomly selected male from the reference colony (C16 for clonal line A; STC6 for clonal line B). To examine data spanning greater genetic distances, we sequenced one male each from two additional stock colonies from each clonal line. Pairwise genetic distances were calculated between diploid females from the respective colonies. Dotted lines depict linear models. The positive slope and near-zero *y* intercepts indicate that, rather than excess crossovers being a quirk of haploid male production, crossovers accumulate gradually over generations of diploid-producing parthenogenesis. Source data

**Extended Data Fig. 4:**
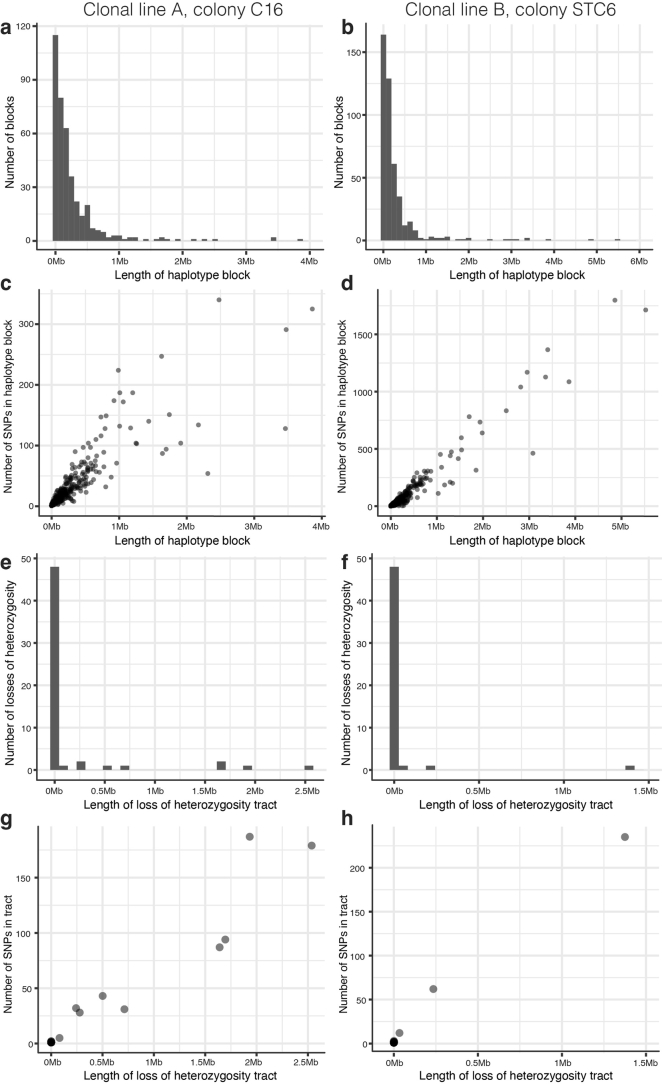
Haplotype blocks and loss of heterozygosity tracts resulting from crossovers and gene conversions within colonies of *O. biroi.* **a**–**d**, The distribution of haplotype block lengths among haploid males and **e**–**h**, the distribution of sizes of loss of heterozygosity among diploid females from a single colony of clonal line A (**a**, **c**, **e**, **g**; colony C16) and clonal line B (**b**, **d**, **f**, **h**,; colony STC6). **a**, **b**, Histograms depict the distance in base pairs between recombination events, or between a recombination event and the end of the contig on which it occurred. In addition to gene conversion events, which span at most a few kb in length, short haplotypes represent remnants of comparatively old recombination events that many subsequent recombination events have since broken up. By contrast, long haplotype blocks likely were formed by relatively recent recombination events and have yet to be broken up by additional recombination events. The fact that many intermediate sizes of haplotypes are found in this dataset suggests that the observed recombination events occurred gradually over many generations. **c**, **d**, Scatterplots depicting the association between the length of haplotype blocks and the number of ancestrally heterozygous SNPs comprising each haplotype block. Each point represents one of the haplotype blocks also depicted in **a** and **b**. Long haplotype blocks inferred to have resulted from crossover recombination were supported by a corresponding number of SNPs. **e**, **f**, Histograms depict the length of loss of heterozygosity tracts or distances between the beginning of a loss of heterozygosity tract and the end of a contig. Note that the total number of tracts of losses of heterozygosity is greater than the number of crossovers reported in the main text because long segmental losses of heterozygosity that spanned multiple contigs likely resulted from a smaller number of crossover events (see Methods). **g**, **h**, Scatterplots depict the number of ancestrally heterozygous SNPs that became homozygous as a function of the length of the respective loss of heterozygosity tract. Each point represents one of the haplotype blocks also depicted in **e** and **f**.

Taken together, these results imply that *O. biroi* uses a previously unknown mechanism to maintain heterozygosity. One possibility is that crossovers do not occur and the putative chiasmata in chromosome squashes result from another kind of DNA entanglement. To explore this possibility, we looked for crossovers using genetic evidence, which can be challenging because, when using short-read sequencing in diploid asexual lineages, they can only be identified using loss of heterozygosity as a proxy (Fig. 2a). However, sequencing haploid genomes allows identification of inherited crossovers regardless of whether heterozygosity was lost. Fortunately, *O. biroi* clonal lines occasionally produce vestigial haploid males, presumably via meioses not followed by central fusion. We randomly sampled several haploid males and diploid females from two clonal lines (line A, colony C16; line B, colony STC6) and sequenced their whole genomes to identify crossovers. This revealed hundreds of crossovers among haploid genomes, which contrasted with only a few crossovers inferred via loss of heterozygosity among diploid genomes (Fig. 2b,c, Extended Data Fig. 4 and Supplementary Data 1; haploids—line A, 346 crossovers and line B, 418; diploids—line A, 8 and line B, 3). This effect was specific to crossover recombination, as haploids and diploids had roughly equal numbers of gene conversion-sized events (haploids—line A, 47 gene conversions and line B, 32; diploids—line A, 49 and line B, 51). Many more crossovers are thus evident by comparing haploid genomes than by observing losses of heterozygosity among diploid genomes.

**Extended Data Fig. 5:**
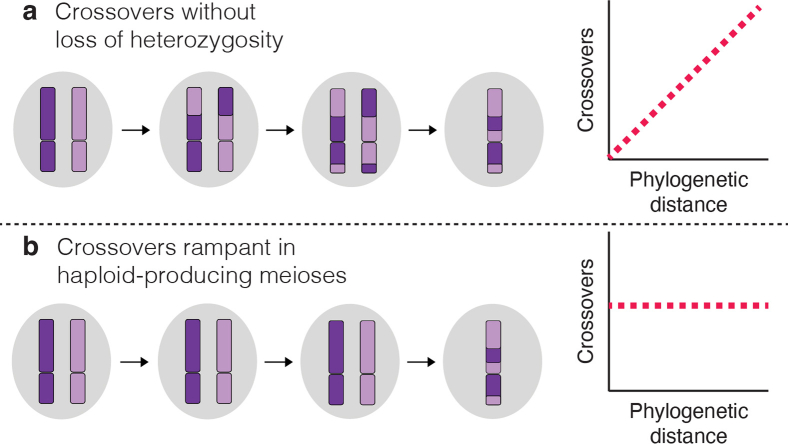
Alternative explanations for the elevated number of crossovers detectable among haploid genomes relative to diploid genomes. Illustrations of alternative explanations for the discrepancy in the number of crossovers detectable in haploid versus diploid genomes and predictions of how the number of crossovers should scale with phylogenetic distance for each explanation. Purple colouring on chromosomes indicates allelic identity. Red dotted lines in plots indicate hypothesized trends for each scenario. **a**, If crossovers occur in typical *O. biroi* meioses that are followed by central fusion and produce diploid offspring, then crossovers accumulate slowly over generations. If true, crossovers would also be present in diploid genomes but are undetectable because heterozygosity is not lost. In this scenario, the number of crossovers detected between pairs of haploids would scale positively with the phylogenetic distance between those individuals and would have a y-intercept close to zero. **b**, If crossovers were absent from typical *O. biroi* meioses, but crossovers were rampant in meioses that are not followed by central fusion (the ‘haploid-producing’ meioses), then similar numbers of crossovers should be detectable among pairs of haploid males, regardless of genetic distance. In this scenario, the number of crossovers detected between pairs of haploids would have a slope of zero and a positive y-intercept.

**Extended Data Fig. 6:**
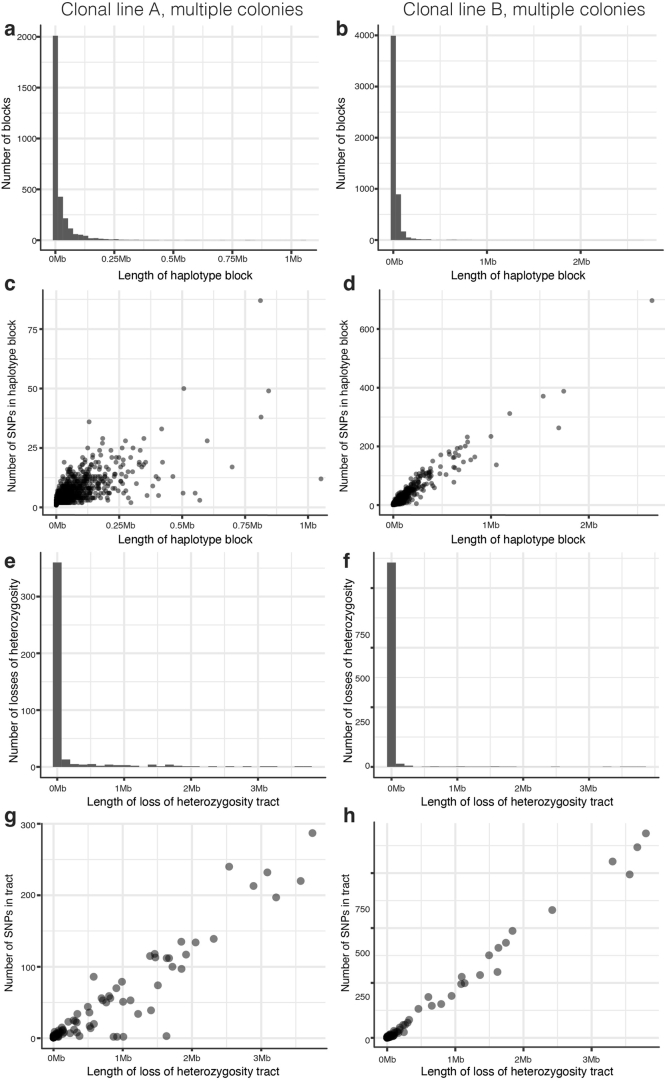
Haplotype blocks and loss of heterozygosity tracts resulting from crossovers and gene conversion across different colonies of *O. biroi.* **a–d**, The distribution of haplotype block lengths among haploid males and **e–h**, of size of loss of heterozygosity among diploid females from multicolony datasets of Line A (**a**, **c**, **e**, **g**) and Line B (**b**, **d**, **f**, **h**). **a**, **b**, Histograms depict the distance in base pairs between recombination events, or between a recombination event and the end of the contig on which it occurred. In addition to gene conversion events, which span at most a few kb in length, short haplotypes represent remnants of comparatively old recombination events, which many subsequent recombination events have since broken up. By contrast, long haplotype blocks likely were formed by relatively recent recombination events and have yet to be broken up by additional recombination events. **c**, **d**, Scatterplots depicting the association between the length of haplotype blocks and the number of ancestrally heterozygous SNPs comprising each haplotype block. Each point represents one of the haplotype blocks also depicted in **a** and **b**. **e**, **f**, Histograms depict the length of loss of heterozygosity tracts, or distances between the beginning of a loss of heterozygosity tract and the end of a contig. Note that the total number of tracts of losses of heterozygosity is greater than the number of crossovers reported in the main text because long losses of heterozygosity that spanned multiple contigs likely resulted from a smaller number of crossover events (see Methods). **g**, **h**, Scatterplots depict the number of SNPs that were ancestrally heterozygous but became homozygous as a function of the length of the respective loss of heterozygosity tract. Each point represents one of the haplotype blocks also depicted in **e** and **f**.

*O. biroi* females do not mate in the laboratory and males are evolutionary dead-ends in that they do not produce offspring. Therefore, since the common ancestor of the studied males, all meioses were followed by central fusion, until each male resulted from a final meiosis not followed by central fusion. This implies two possible alternative explanations of the dramatic differences in observed versus inferred crossover events between males and females, respectively (Extended Data Fig. 5). First, the meioses that produce haploid offspring might not only lack the subsequent central fusion but also differ from typical meioses in other ways. Specifically, our data could be explained if typical meioses lacked crossovers but the haploid-producing meioses featured excessive recombination. Alternatively, the crossovers observed among haploid male genomes might have accumulated over many preceding generations of females. In that case, crossovers should also be present but ‘hidden’ in diploid female genomes because no heterozygosity was lost. To distinguish between these scenarios, we sampled individuals from different colonies within each clonal line, sequenced their genomes and identified crossovers between all pairs of samples. This multicolony dataset confirmed our initial results, also revealing many more crossovers among haploid than among diploid genomes (Extended Data Fig. 6 and Supplementary Data 2). We then analysed how the number of crossovers among haploid males scaled with phylogenetic distance. If haploid-producing meioses featured excessive recombination, then we would expect non-zero *y* intercepts because closely related males would differ from each other by many recombination events. However, for both clonal lines, the *y* intercepts were approximately zero (Fig. 2d; line A, 0.007 and line B, 0.004), implying that haploid males do not arise from meioses with exceptional numbers of crossovers. Moreover, the number of crossovers between males was tightly correlated with genetic distance (line A—*R*^2^ = 0.94, *P* = 0.007; line B—*R*^2^ = 0.99, *P* = 0.002), revealing that crossovers had accumulated over many generations and thus must occur regularly without loss of heterozygosity in *O. biroi* meiosis.

**Fig. 3:**
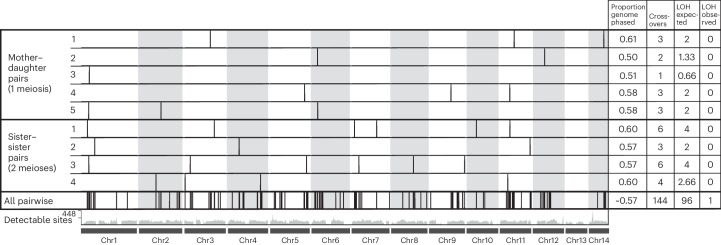
Crossovers occur every meiosis without loss of heterozygosity. Karyoplot depicting crossovers (tick marks) inferred between each pair of phased genomes from linked-read sequencing data and all pairwise comparisons (All pairwise) between the different families of unknown pedigree. The numbers of sites at which crossovers are detectable per 200 kb window are shown as a grey histogram (Detectable sites). For each pair, we show to the right of the karyoplot the proportion of the genome phased in both samples (that is, the proportion of the genome for which crossovers are detectable), the total number of crossovers detected, the number of losses of heterozygosity expected and the number of losses of heterozygosity observed. Assuming Mendelian segregation, two-thirds of inherited crossovers should be associated with loss of heterozygosity but, surprisingly, only one of the observed crossovers was. Source data

**Extended Data Fig. 7:**
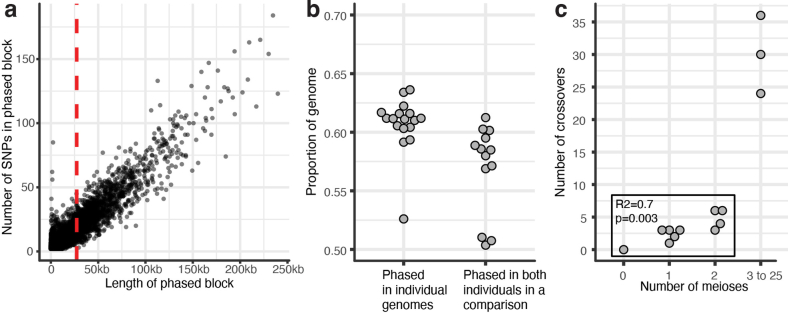
Phasing linked-read sequencing libraries to detect crossovers. **a**, The relative phasing efficiency for a single example individual library (LinkedReadPedigree3_Daughter_1) is given by a scatterplot depicting the association between the length of phased blocks and the number of ancestrally heterozygous SNPs per block, with the red line representing the phased block N50. **b**, The proportion of the genome that was successfully phased is given for all 17 individual linked-read genomes and for all 13 pairs of individuals compared for initial crossover detection. **c**, Scatterplot depicting the association between the number of crossovers detected and the number of intervening meioses for each pair of genomes. Zero meioses separate two libraries prepared from the same individual (one negative control); one meiosis separates mothers and daughters in five pairs and two meioses separate sisters in four pairs. The pedigree was not known for three ‘positive control’ comparisons, but the number of intervening meioses falls between 3 and 25 (see Methods). The correlation between the number of crossovers and the number of meioses for all known-pedigree comparisons (box) was analysed using linear regression, with the significance tested using an F-test (p = 0.003).

To confirm that crossovers occur without segmental loss of heterozygosity, we need to compare haplotypes from known pedigrees. Because haploid males occur too rarely to be used in pedigree studies, we used linked-read sequencing to obtain haplotype-resolved diploid genomes of five mother–daughter pairs (one meiosis each) and four sister–sister pairs (two meioses each). For each individual, we sequenced the linked-read library to at least 50× coverage with Illumina, allowing us to phase 61% of the genome on average, with an average phased block N50 of 27 kilobases (kb) (Extended Data Fig. 7 and Supplementary Table 2). By comparing pairs of phased genomes, we can detect crossovers in heterozygous regions that are reliably phased in both samples (55% of the genome on average) and for which at least one recombined chromatid has been inherited. Crossovers are not detectable if neither recombined chromatid has been inherited, which is expected for one in four crossovers (Fig. 1b). We detected crossovers in all known-pedigree pairs, with an average of 2.38 crossovers per meiosis (Fig. 3 and Supplementary Table 3; 31 crossovers sampled across the 13 meioses from known-pedigree pairs). Crossovers were distributed across all chromosomes, except for the heteromorphic chromosome 13, for which pervasive structural variation severely limits crossover detection^27^. Adjusted for underestimation due to incomplete phasing and non-detection of uninherited crossovers (Methods), these data suggest that, on average, 5.5 crossovers occur in each meiosis (0.4 crossovers per chromosome, 95% confidence interval (CI) 0.3–0.49). This is probably an underestimate because recombination hotspots might fall within regions that have ancestrally lost heterozygosity and non-crossover products might disproportionately be co-inherited. Regardless, our data show that crossovers occur in every meiosis.

None of the 31 identified crossovers in this analysis was associated with segmental loss of heterozygosity, although 15 were associated with gene conversion (Fig. 3 and Supplementary Table 3). This is surprising because, under Mendelian inheritance, we expect two-thirds of inherited crossovers to be associated with segmental loss of heterozygosity (Fig. 1b). To investigate whether this pattern held for larger numbers of crossovers, we selected one individual from each pedigree and performed all pairwise comparisons of their phased genomes (seven different pedigrees with 21 pairwise comparisons). Pairs of genomes in this analysis were separated by at least three but fewer than 25 meioses. This produced a total of 144 crossovers (including those that were detected in known-pedigree pairs). Of these, 143 crossovers occurred without segmental loss of heterozygosity, while one resulted in a >200 kb loss of heterozygosity tract (Fig. 3, Supplementary Table 3 and Supplementary Data 3 and 4). Thus, both crossover products were inherited for all but one detected crossover. This is a statistically significant departure from the expectation that segmental loss of heterozygosity should occur for two-thirds of inherited crossovers (binomial test, *P* < 0.0001). The only other changes in heterozygosity were small (≤466 bp) losses of heterozygosity consistent with gene conversion (Extended Data Table 2, Supplementary Table 3 and Supplementary Data 3). This high-fidelity heterozygosity maintenance is showcased by sliding window analyses, which reveal remarkable stability of genome-wide heterozygosity (Extended Data Table 1). From a per-offspring perspective, loss of heterozygosity is also very rare; of the 15 offspring sampled (from both linked-read and short-read sequencing), none had a segmental loss of heterozygosity (95% CI 0–0.2 for the true proportion of offspring that incur a segmental loss of heterozygosity).

Although the rare large losses of heterozygosity might often be detrimental in *O. biroi*, at least some are tolerated and inherited. This is illustrated by several cases in which multiple individuals per colony carried the same loss of heterozygosity (Supplementary Data 1–4). Although most known losses of heterozygosity do not have discernible phenotypic effects, some can dramatically alter the ants’ appearance, as in the case of a recently described ‘queen-like mutant’ that resulted from a loss of heterozygosity spanning 2.25 megabase pairs (Mbp) on chromosome 13 (ref. ^27^).

### Reciprocally recombined chromatids are co-inherited

**Fig. 4:**
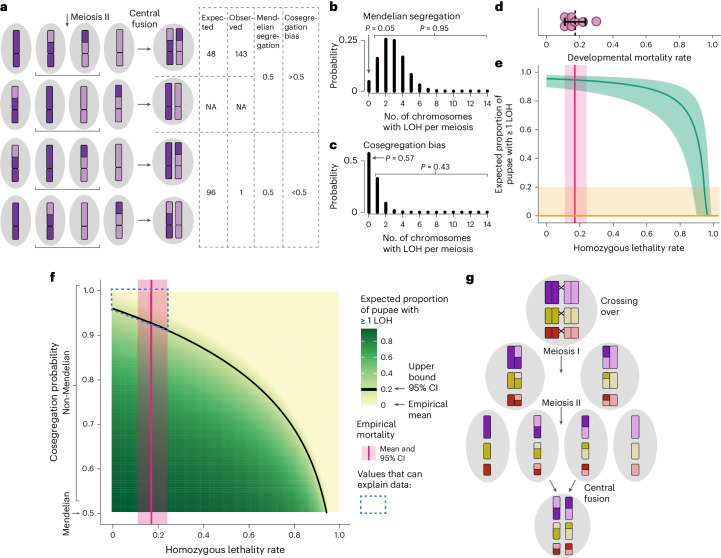
Non-Mendelian cosegregation of crossover products maintains heterozygosity. **a**, Cartoon depicting the four possible segregation patterns following a crossover. Shown are the stage following meiosis II, at which there are four haploid pronuclei and the stage following central fusion, when the diploid zygote is formed. For each pattern, the expected and observed numbers of crossovers detected by linked-read sequencing are shown. Crossovers cannot be detected if neither crossover product was inherited, so the numbers for that outcome are listed as not available (NA). Under Mendelian segregation, the probability of loss of heterozygosity is 0.5 but such values are incompatible with our results. If crossover products cosegregate (Cosegregation bias) such that they are always inherited together, heterozygosity would rarely be lost. **b**,**c**, Probability mass functions of binomial models depicting the number of chromosomes expected to incur a loss of heterozygosity per meiosis, assuming the adjusted empirical crossover rate of 5.5 crossovers per meiosis. **b**, The model assumes Mendelian segregation. **c**, The model assumes a cosegregation probability of 0.9 (chosen because, at this probability, it is more likely for heterozygosity to be lost for zero chromosomes than for one or more chromosomes). Above the plots are the probabilities that losses of heterozygosity occur on zero chromosomes or on one or more chromosomes. **d**, Developmental mortality, shown as the proportion of individuals that died between the early egg stage and pupation in seven replicate colonies (magenta dots); mean (dotted line) and 95% CI (black bar). **e**, Theoretical model depicting the ability of homozygous lethality to maintain heterozygosity if segregation is Mendelian. Shown in green is the expected proportion of offspring with a loss of heterozygosity on one or more chromosomes (from the binomial model depicted in **b**) minus the homozygous lethality. Values based on the mean empirical crossover rate are shown as a dark line and values based on the 95% CI are shown in green shading. The empirical developmental mortality rate (which is the hypothetical maximum homozygous lethality) is shown in magenta (mean depicted by dark line, 95% CI depicted by shading), revealing that at least 86% of offspring should bear a loss of heterozygosity. The empirical proportion of offspring with at least one loss of heterozygosity is shown in orange (mean depicted by dark line, 95% CI depicted by shading), revealing that developmental mortality rates would need to be at least 0.87 for homozygous lethality to produce proportions that fall within the observed range. Thus, homozygous lethality can have at most a small effect on heterozygosity maintenance. **f**, Heatmap depicting the proportion of offspring expected to bear a loss of heterozygosity on one or more chromosomes. ‘Reasonable’ parameter combinations can be found above the black line, which depicts proportions of 0.2, the upper bound of our 95% CI for proportions of offspring that bear one or more loss of heterozygosity. The empirical developmental mortality rate (the hypothetical maximum homozygous lethality) is shown in magenta. Therefore, the only parameter combinations that can explain the degree of heterozygosity maintenance observed in this study are found within the blue dashed outline, revealing that a cosegregation probability >0.91 occurs in *O. biroi*. **g**, Heterozygosity is maintained via a programmed violation of Mendelian segregation, whereby crossover products cosegregate such that both are inherited. Source data

These data cannot be explained via Mendelian inheritance of crossover products (Fig. 4a). Deviations from Mendelian inheritance can result from mechanisms acting at different points in an organism’s life cycle. Because crossovers are non-randomly inherited in *O. biroi*, the underlying mechanism must operate after homologous chromosomes separate (in anaphase I of meiosis) and could occur as chromosomes segregate during meiotic divisions. Under Mendelian inheritance, the segregation of one crossover product does not depend on the reciprocal crossover product. If this were the case in *O. biroi*, losses of heterozygosity would be the rule; even with our conservative empirical estimate of 0.4 crossovers per chromosome per meiosis, binomial modelling shows that, if segregation were Mendelian, 95% of offspring (95% CI 90%–98%) would bear a loss of heterozygosity on one or more chromosomes (Fig. 4b). Therefore, chromosomal segregation must be non-random, such that if one crossover product is included in a central pronucleus, then the reciprocal crossover product is found in the other central pronucleus (Fig. 4a). Such cosegregation of crossover products would ensure that inheritance of both crossover products is the rule, whereas inheritance of one crossover product and one non-crossover product is a rare exception (Fig. 4a,c).

The only possible alternative to crossover cosegregation would be that losses of heterozygosity are lethal. Such ‘homozygous lethality’ is thought to maintain heterozygosity in other parthenogenetic species, including the cape honeybee, which also reproduces via automixis with central fusion^18,19^. Because egg laying precedes meiotic divisions and central fusion in *O. biroi*^9^, homozygous lethality would occur after eggs are laid but before the pupal stage, when we measured loss of heterozygosity. To measure developmental mortality, we used our transgenic marker line to track survival from the egg to the pupal stage in seven replicate colonies (Extended Data Fig. 3). The mean proportionate mortality was 0.17 (95% CI 0.11–0.24) (Fig. 4d). We then adjusted our binomial model, making the strict assumptions that (1) all developmental mortality results from loss of heterozygosity and (2) all losses of heterozygosity are lethal. This revealed that mortality rates of at least 0.87 would be required to produce proportions of offspring with loss of heterozygosity that fall within the 95% CI of our empirical estimate (Fig. 4e). By contrast, our observed developmental mortality rate predicts expected proportions of offspring bearing loss of heterozygosity to be at least 0.86 under Mendelian segregation (Fig. 4e). Therefore, even under our conservative estimate of crossover rates, homozygous lethality alone cannot maintain heterozygosity in *O. biroi*. Instead, the mechanism underlying non-Mendelian inheritance must act before the offspring genotype is determined (when central pronuclei fuse to form the diploid zygotic nucleus). Thus, crossover product cosegregation bias is required to explain our data.

To estimate the strength of this bias, we varied the probability of crossover product cosegregation as a parameter in our binomial model. Because we cannot rule out a role for homozygous lethality, we calculated the expected proportion of offspring with loss of heterozygosity for a range of homozygous lethality rates and cosegregation probabilities. Taking the upper bound of the developmental mortality rate as the hypothetical maximum homozygous lethality, we find that the crossover product cosegregation probability must be at least 0.91 for heterozygosity to be maintained to the extent observed (Fig. 4f). This is an underestimate of the actual cosegregation bias because some developmental mortality probably stems from factors other than homozygosity and because we may have underestimated the true crossover rate. Thus, strongly non-Mendelian cosegregation of crossover products maintains heterozygosity in *O. biroi*.

## Discussion

Understanding extreme modes of reproduction can provide unexpected insights into fundamental cellular processes. Here we investigated a parthenogenetic ant that produces diploid offspring by fusing two haploid pronuclei from a single meiosis (Fig. 1a). This reproductive mode should lead to segmental loss of heterozygosity distal to crossovers but we show that such loss of heterozygosity is exceedingly rare (Figs. 1e and 3). Surprisingly, we found that crossovers occur every meiosis but heterozygosity is maintained because both crossover products are regularly co-inherited (Figs. 2 and 3).

It is tempting to ask what advantages the strategy used by *O. biroi* might have over parthenogenetic strategies that lack crossing over. There are certainly differences—the recombination of haplotypes, meiotic gene conversions and rare losses of heterozygosity mean that *O. biroi* should accumulate genetic diversity faster than apomictic (mitotic-like) parthenogenetic lineages in which homologous chromosomes do not recombine^2^. However, when a parthenogenesis-inducing mutation arises, it does not compete against possible alternative parthenogenetic strategies but against the ancestral, sexual mode of reproduction. Therefore, there need not be an absolute advantage of one parthenogenetic strategy over other modes for one mode to be successful in any particular case. Female-producing parthenogenesis has evolved many times in the Hymenoptera and the cytological mechanism is almost always automictic (meiotic) rather than apomictic, with all described automictic species retaining crossovers^6,28^. One possibility is that it is simply mechanistically easier to evolve automixis rather than apomixis. Similarly, developmental constraints such as the requirement of crossovers for accurate chromosomal segregation in many organisms might impede the evolution of achiasmate meiosis.

We found that reciprocal crossover products cosegregate with a probability of at least 0.91, an 800% increase over the Mendelian expectation (Fig. 4f). Because our methods underestimate the crossover rate, the true cosegregation probability may be considerably higher. The best-known form of non-Mendelian segregation is selfish meiotic drive^16^, in which driving alleles promote their own transmission over alternative alleles on the homologous chromosome. However, *O. biroi* demonstrates non-Mendelian segregation across all chromosomes without allele-specific bias. Therefore, this phenomenon may result from a systemic perturbation of the meiotic programme, where cosegregation of crossover products leads to the programmed inheritance of both alleles at all loci (Fig. 4g). Alternatively, this biased transmission of crossovers to the central pronuclei may reflect a previously overlooked aspect of normal meiosis.

Indeed, meiotic drive for crossover status, rather than allelic identity, occurs in meiosis II of human oogenesis, where crossover products are nearly twice as likely as non-crossover products to end up in the oocyte rather than the second polar body^29^. As in *O. biroi*, this phenomenon occurs on many chromosomes and, because no allele has biased transmission, it has been referred to as ‘unselfish’ meiotic drive^30^. Despite this moniker, biased transmission of crossover products could, in principle, be exploited. For example, if the biased transmission of recombined chromatids is mediated by an epigenetic mark, selfish alleles could recruit that mark in the absence of recombination and thereby further their own transmission^30^. We note, however, that if such an allele were to arise in an *O. biroi* clonal line, it would not be able to spread sexually throughout the population.

A recent report describes heterozygosity maintenance despite regular crossing over (described as ‘cosegregation of recombinant chromatids’) in the asexual nematode *Mesorhabditis belari*^31^. This represents another case of unselfish meiotic drive for crossover status. On the surface, the genetic inheritance systems of *O. biroi* and *M. belari* are very similar and it is remarkable that two highly diverged species convergently evolved both asexual reproduction and non-Mendelian segregation. On the other hand, it is unclear whether similar molecular mechanisms are involved. In *M. belari*, meiosis I is abortive^31^, whereas *O. biroi* meiosis features complete meiosis I and II divisions before central fusion^9^. Although the mechanisms may differ, crossover cosegregation in both *O. biroi* and *M. belari* requires cellular memory of crossovers, reminiscent of the unselfish meiotic drive for crossover inheritance in human oogenesis.

How do cells remember crossovers? One possibility is that delayed or incomplete resolution of crossover recombination intermediates facilitates lingering connections between recombined chromatids. Such connections might tether chromatids together through meiotic divisions and bias the orientation of recombined products to particular pronuclei, thus facilitating the inheritance of recombined chromatids. Although such a mechanism is currently not known to direct segregation based on recombination, chromatin threads formed from heterochromatin have been shown to mediate meiotic segregation in both plants and *Drosophila melanogaster*^32–34^. These connections might be difficult to detect, as is the case for ‘ultrafine DNA bridges’, which are not detectable with traditional DNA stains and sometimes result from homologous recombination^35^. Alternatively, epigenetic marks associated with crossover recombination^36^ might facilitate biased orientation of recombined chromatids during anaphase. Future molecular studies of *O. biroi* oogenesis may elucidate the mechanism underlying crossover co-inheritance.

The unselfish meiotic drive for recombination status in human oogenesis illustrates that biased segregation of crossovers can also occur in sexual species, even if the segregation bias is much weaker than what we report here for *O. biroi*. Given that very few studies have had the ability to detect non-random inheritance of crossovers, this raises the possibility that cellular memory of crossovers is a poorly understood yet widely spread aspect of ‘normal’ chromosomal segregation. Thus, we may have been missing a key mechanism that selection can act on to tune recombination rates. This mechanism can then be elaborated on in extreme reproductive systems such as parthenogenesis.

## Methods

### Chromosome squashes

To study chiasmata in *O. biroi* meiosis, we produced squashes of meiotic nuclei at metaphase I. For this, we slightly modified the method of ref. ^37^. Hymenopteran oocytes are arrested at metaphase I before egg laying^38,39^, so we dissected ovaries in 1× PBS, targeting the most mature oocytes available and incubated oocytes in hypotonic solution (1% sodium citrate) on a poly-l-lysine coated glass slide for 12 min. Next, we removed the hypotonic solution and replaced it with 10 µl of 45% acetic acid/1% PFA/1× PBS for 6 min. We then physically squashed oocytes with a glass coverslip and flash-froze the slide/coverslip in liquid nitrogen. Immediately after removal from liquid nitrogen, we removed coverslips with a razor blade and incubated slides in cold 3:1 methanol:glacial acetic acid for 10 min. We rinsed slides twice in 1× PBS and then dehydrated them for 5 min each in 70%, 80% and 100% ethanol (all ice-cold) before air-drying for at least 24 h.

We stained slides with DAPI (1 µg per ml) for 10 min before air-drying, then added SlowFade Diamond mounting medium, added the coverslip and sealed with nail polish. We kept mounted slides at room temperature overnight before imaging and subsequently stored them at 4 °C.

We performed confocal microscopy using Zen image acquisition software on a Zeiss LSM 900 with the 405 nm laser line. Images were obtained with Airyscan in super-resolution mode using a Zeiss LD LCI Plan-Apochromat ×40/ 1.2 NA objective lens and Zeiss Immersol Sil 406 immersion medium. We performed deconvolution using Huygens Professional v.22.04 (Scientific Volume Imaging, http://svi.nl) with default parameters. *Z*-projection images were produced from stacks taken at 0.18 µm steps using ImageJ/FIJI^40^.

### Probability of segmental loss of heterozygosity

To estimate the number of chromosomes expected to lose heterozygosity each meiosis, we used a binomial model assuming one crossover per chromosome (higher numbers of crossovers per chromosome produce a higher probability of loss of heterozygosity, meaning that, if anything, our calculations should underestimate loss of heterozygosity rates). Because all pairs of homologous chromosomes assort independently, each chromosome can be considered an independent trial (*n* = 14 chromosomes in the *O. biroi* karyotype). Chromatids are expected to segregate randomly in meiosis II, so in central fusion automixis there is a probability of 0.5 of crossover-induced loss of heterozygosity on any one chromosome (Fig. 1b). We used the dbinom() function in R v.4.0.5 (ref. ^41^) to calculate the binomial probability mass function for losing heterozygosity on all possible numbers of chromosomes in any given meiosis. To estimate the proportion of offspring expected to have a loss of heterozygosity, we took the sum of the probabilities that loss of heterozygosity occurs on one or more chromosomes.

To fit this model using empirical data, we substituted the empirical crossover estimate obtained from linked-read sequencing (‘Code availability’). To explore the potential of cosegregation bias (the possibility that two or zero crossover products are inherited more commonly than one crossover product and one non-crossover product) to explain heterozygosity maintenance, we varied the probability of loss of heterozygosity following a crossover between 0.5 (Mendelian) and 0 (complete cosegregation bias). To explore the degree to which homozygous lethality might help maintain heterozygosity, we subtracted hypothetical developmental mortality rates ranging from 0 to 1 from the proportion of offspring expected to have a loss of heterozygosity, making the conservative assumptions that (1) all mortality comes from loss of heterozygosity and (2) any loss of heterozygosity is lethal. To determine which parameter sets of these values are likely to reflect reality, we compared these calculations to empirically determined values for the rate of developmental mortality and the proportion of offspring with segmental losses of heterozygosity.

### Generation of the transgenic marker line

To track pedigrees of ants within colonies, we generated a transgenic line with a stable fluorescent label. For this, we used a plasmid with a piggyBac backbone to transpose the baculovirus-derived ie1 enhancer/promoter element to drive expression of the red fluorescent protein dsRed (Extended Data Fig. 2). This plasmid (pBac-ie1-dsRed) was assembled as part of a previous study^42^. For transgenesis, we collected 660 eggs from clonal line B^12,26^, injected them with pBac-ie1-dsRed and reared the resulting fluorescent individuals according to our established protocol^42^, yielding isogenic colonies of fluorescent individuals derived from single founders. The results from the injection experiments are summarized in Supplementary Table 1 and the procedure for generating and rearing transgenic ants is outlined in Extended Data Fig. 2.

The dsRed fluorescence in the transgenic line is detectable starting in the first larval instar and remains visible throughout metamorphosis and in adults (Extended Data Fig. 2). To reduce the movement of animals during imaging, we placed them at −20 °C for 5 min beforehand. In larvae, fluorescent dots are initially visible on each body segment. The dsRed fluorescence becomes diffusely visible throughout the body during the final larval instar. This fluorescence remains visible after pupation. Pigmentation of the cuticle partially masks fluorescence in older pupae but fluorescence is still apparent at the tip of the gaster, joints and other areas. Fluorescence remains detectable in live adults >6 months old.

To characterize the fluorescent phenotype more closely, we analysed the fluorescence of five transgenic and five wild-type individuals, each removed from cycling colonies ~1 month after eclosion. We placed individuals in ethanol for 10 s before dissecting them in PBS. We dissected brains and cut bodies between the thorax and gaster. We fixed brains and gasters in 4% PFA for 30 min, washed them 3× in PBS with 0.1% Triton and mounted them in Dako mounting medium. We detected dsRed and autofluorescence by imaging brains and gasters using the red and green fluorescence channels of an epifluorescence microscope (Extended Data Fig. 2). While individuals from a previous study with [ie1-dsRed, ObirOrco-QF2, 15xQUAS-GcaMP6s] express dsRed in their antennal lobes^42^, fluorescence is not visible in dissected brains of ants with ie1-dsRed alone. This indicates that the ie1 promoter does not normally drive expression in brain tissue but interaction between adjacent transgenes can lead to leaky expression^42^. The brightest dsRed fluorescence was detected in the gaster. We observed intense dsRed fluorescence in the poison gland filament, the Malpighian tubules and structures associated with the fat body (Extended Data Fig. 2). We reared a large population of a single transgenic line, which readily forms functioning colonies and produces offspring with stable transgene expression, indicating that the individuals are fully viable.

The transgenic ants developed for this study are available on request, depending on availability and assuming that all regulatory requirements are met.

### Fitness of the transgenic marker line

To assess the possibility of a fitness deficit due to the transgenic insertion, we tested whether the transgenic ants had reduced reproductive output compared to wild-type ants of the same clonal line. We calculated the proportion of offspring with dsRed fluorescence from colonies (*n* = 17) containing a single transgenic regular worker and 19 wild-type ants. We took the mean proportion of transgenic offspring across two colony cycles and tested whether the value was less than the expected proportion of 0.05 (1/20 transgenic ants in each colony, with most wild types being regular workers rather than intercastes). The mean dsRed offspring proportion was 0.11 ± 0.54 s.d., not significantly less than the expected 0.05 (Extended Data Fig. 3; *P* = 0.999, one-tailed one-sample Wilcoxon test). The transgenic line, therefore, does not have reduced fitness compared to wild-type ants from the same clonal background.

### Genomic characterization of the transgenic marker line

We counted and mapped the position of the transgene insertion as described previously^42^. Briefly, we investigated the genome of one of the samples sequenced from a known pedigree (see below for sequencing details). We aligned reads to the *O. biroi* reference genome^11^ and separately to the transgene sequence. We recorded read depth of well-assembled genomic regions using samtools depth -aa^43^ and compared the read depth of the transgene insert to read depths at randomly selected trusted diploid sites. The mean read depth of the transgene insert was ~0.5× that of the inferred genome-wide average. Because these sequences derive from diploid *O. biroi* workers, we conclude that the transgene is present in only a single copy (Extended Data Fig. 3). As expected for piggyBac, the backbone was not incorporated into the genome.

To map the genomic locus containing the transgene insertion, we identified ‘junction reads’ that aligned to both the *O. biroi* reference genome and the transgene insert^44,45^ using the Integrative Genomics Viewer^46^ and queried alignments by each junction read name using ‘samtools view’^43^. We used CLUSTAL 2.1 in the R package msa^47,48^ to perform several sequence alignments of the junction reads and generate consensus sequences. We used BLAST^49^ to search the reference genome for the consensus sequences, which identified the same position the junction reads had aligned to. The position identified contained a TTAA motif, which is expected at the piggyBac transposon insertion site^50–53^. The insertion was located between positions 8810280 and 8810281 on the ninth chromosomal scaffold, which is not within any predicted gene model (Extended Data Fig. 3).

### Ant rearing and obtaining mother–daughter pairs

To study the amount of crossover recombination and loss of heterozygosity per meiosis, we needed to sequence genomes of ants from known pedigrees. To obtain known-pedigree pairs, we created experimental colonies consisting of a single focal fluorescently labelled transgenic ant with 19 wild-type ants. After colonies laid eggs and reared them to the pupal stage, we sampled red fluorescent individuals of known pedigree (mother–daughter and sister–sister pairs). Individuals from different pedigrees were sometimes compared to sample larger numbers of meioses. In these cases, we did not know the exact pedigree but, because the ants were from a single transgenic line recently derived from a single founding individual, we knew the range of possible numbers of meioses separating each pair of individuals.

Haploid males were serendipitously collected from several stock colonies whenever they were found. To make the data as comparable as possible, we haphazardly sampled diploid females from the same stock colonies.

### Short-read whole-genome shotgun sequencing

We performed whole-genome shotgun sequencing on 22 Illumina DNA libraries. For each library, we disrupted an individual ant (adult or pupa) using a Qiagen TissueLyser II and extracted genomic DNA using Qiagen’s QIAmp DNA Micro Kit. We prepared libraries using Illumina’s Nextera DNA Flex kit and targeted 40× sequencing coverage for each library to ensure sufficient coverage to detect heterozygosity accurately. Reads were trimmed with Trimmomatic 0.36 (ref. ^54^), aligned with bwa mem^55^ to the *O. biroi* reference genome (Obir_v5.4, GenBank assembly accession: GCA_003672135.1) and subsequently sorted, deduplicated and indexed with picard^56^. Variants were called with GATK HaplotypeCaller (v.4.2)^57^ and filtered using the hard-filtering recommendations of GATK.

We performed additional filtering to include only high-confidence variants in this analysis. We excluded falsely collapsed regions in the reference genome by filtering against sites found to be heterozygous in haploid males, variants with three or more alleles in a single diploid clonal lineage and sites with read depth greater than twice the genome-wide mean. To exclude erroneous calls of heterozygosity or homozygosity, we filtered against sites with read depth <15, with proportionate minor allelic depth <0.25 in putatively heterozygous samples or with non-zero minor allelic depth in putatively homozygous samples. While performing data analysis, we observed that forgoing any of these additional filtering steps led to many false-positive gains of heterozygosity, losses of heterozygosity and recombination events.

### Loss of heterozygosity in known pedigrees

To analyse mother–daughter pairs, we classified all differences in genotype as loss of heterozygosity or gain of heterozygosity. Point-mutation-induced gains of heterozygosity were inferred at sites where an individual was heterozygous and possessed an allele found in no other members of that clonal lineage. To screen against remaining false positives, we visually inspected all putative changes in heterozygosity by loading aligned reads in IGV2.6.2 (ref. ^46^) to remove all sites with ambiguous heterozygosity calls (that is, putatively homozygous sites with one or more aligned reads bearing the alternate allele) or with signatures of assembly errors (variants with reads indicative of heterozygosity in haploid males). Viewing alignment files in this way included reads GATK had screened before genotype calling and such reads often contradicted genotype calls at sites with putative changes in heterozygosity. For a global assessment of heterozygosity differences, we constructed non-overlapping 10 kb windows (21,784 in total) and, for each sample, recorded the proportion of such windows that contained at least one heterozygous SNP.

### Comparison of recombination among haploids and loss of heterozygosity among diploids

To analyse recombination among samples of unknown pedigree, we first inferred ancestrally heterozygous sites following ref. ^9^. For each clonal line, we considered sites heterozygous in any diploid to be ancestrally heterozygous as long as (1) all other diploids had identical heterozygous genotypes or had genotypes homozygous for one of the alleles found in the heterozygous genotype and (2) all haploids were hemizygous for one of the two alleles. Homozygous genotypes at ancestrally heterozygous sites were classified as resulting from loss of heterozygosity. Contiguous runs of such SNPs >1,500 bp in length were classified as losses of heterozygosity resulting from crossovers, whereas shorter stretches were classified as gene conversion tracts. Changing this threshold to 5,000 bp (the approximate upper bound of non-crossover maximum tract length in *D. melanogaster*^23^) did not qualitatively affect the results (that is, the ratio of crossovers detected among haploids to crossovers inferred among diploids changed from 43.3 to 42.3 for line A and from 139.3 to 136.3 for line B). We directly observed recombination events among the genomes of haploid males by recording changes in relative phasing among samples at ancestrally heterozygous sites.

The lengths of haplotype blocks (contiguous tracts of SNPs with identical relative phasing) were recorded as the number of base pairs between the first SNP and last SNP in each haplotype block. To ensure that gene conversion events did not inflate the number of crossovers detected, we removed gene conversion-sized events from the dataset before counting crossover recombination events. For the small number of losses of heterozygosity among diploid females that resulted from crossovers, we counted crossover events only at sites for which the transition from heterozygosity to homozygosity was visible, in accordance with how crossovers were inferred among male haplotypes.

To examine the correlation of crossover recombination with genetic differentiation across colonies, we calculated genetic distance via identity by state at ancestrally heterozygous SNPs using the R package SNPRelate^58^. To ensure statistical independence of comparisons, we did not perform all possible pairwise comparisons but instead compared all samples to a single reference individual. From each reference colony (line A, C16 and line B, STC6), we arbitrarily selected a single diploid female and haploid male as reference individuals for comparison. We counted the number of recombination events between each individual and the reference male and calculated the genetic distance between each individual and the reference diploid female. Using diploid genotypes to calculate genetic distance ensured statistical independence from the recombination events evident among haploid genomes. Because the ability to detect crossovers that have occurred over many generations scales positively with marker density and the clonal lines differed in the number of ancestrally heterozygous SNPs, we normalized the crossover counts for each pair by dividing the number of crossovers detected by the number of ancestrally heterozygous SNPs in that clonal line.

### Linked-read sequencing

To determine whether crossovers occur every meiosis, we performed linked-read whole-genome shotgun sequencing on 17 libraries. To extract high-molecular-weight DNA, we used Qiagen’s MagAttract kit after grinding individual ants (adult or pupal stage) under liquid nitrogen using an up-and-down motion with a plastic pestle. We prepared TELL-Seq^59^ (Universal Sequencing Technologies; Library Prep Kit STD4) libraries according to the manufacturer’s instructions, using 2 ng of input DNA, 15 µl of TELL beads and ten amplification cycles. We targeted at least 60× genome-wide sequencing coverage for each library to ensure reliable phasing.

We performed initial data analysis using pipelines available from Universal Sequencing Technologies. We aligned and ‘linked’ reads using Tell-Read and performed variant calling and phasing using Tell-Sort^59^. This pipeline uses bwa to align reads to the reference genome, calls variants using GATK HaplotypeCaller and phases reads using HapCUT2 (ref. ^60^). We removed all sites with genotype quality <99 or phasing quality <100. We next excluded falsely collapsed regions in the reference genome by filtering against sites found to be heterozygous in haploid males, variants with three or more alleles in a single clonal lineage and sites with read depth <20 or greater than twice the genome-wide mean.

### Analysis of recombination among linked-read libraries

Putative recombination events were identified between phased vcf files using vcftools –diff-switch-error,^61^ which produces a list of all pairs of consecutive SNP positions between which the phasing differs. By comparing phased diploid genomes, we can only confidently infer recombination events in genomic regions reliably phased in both focal samples. Therefore, we screened out putative recombination events that could not be unambiguously confirmed. We removed putative events in which the two focal SNPs were intervened by, or were flanked by, signatures of poor genome assembly. These included sites with no aligned reads in one or more samples, poor mapping quality (aligned reads with MAPQ = 0) or signatures of falsely concatenated genomic regions (coverage greater than twice the genome-wide average or variants observed to be heterozygous in haploid males). To rule out erroneously phased genomic regions, we required that the haplotypic phase remain consistent for at least two SNPs upstream and downstream of putative recombination events. We visually inspected all putative recombination events in IGV.

To determine the proportion of the genome for which crossovers were detectable, we identified all possible pairs of contiguous SNPs and determined whether that pair would have been eliminated by one or more of the above filtering criteria in each pair of samples. Then, we took the proportion of base pairs that fell between retained pairs of SNPs as the proportion of the genome for which crossovers were detectable. For the 14 pairs of samples, this proportion fell between 0.50 and 0.61, indicating that the observed number of crossovers from this analysis is an underestimate by at least 39%.

### Developmental mortality estimates

One mechanism to maintain heterozygosity would be the mortality of individuals with losses of heterozygosity. Such mortality would have to occur after eggs are laid (the meiotic divisions and subsequent central fusion occur following egg laying in *O. biroi*). Measuring developmental mortality requires tracking the survival of known numbers of eggs in replicate colonies, which is complicated by the fact that *O. biroi* are all reproductively totipotent and thus, the caregiver ants could lay additional eggs which would lead to underestimates of developmental mortality. To avoid this complication, we created seven replicate colonies in Petri dish nests with moistened plaster of Paris floors, each with 46 fluorescently labelled transgenic eggs, 40 non-transgenic adults and 38 non-transgenic pupae (pupae were included so that developing larvae could drink their secretions^62^; these pupae eclosed as adults during the experiment). We allowed the adults to rear the eggs and once the eggs had hatched as larvae, we fed the colonies daily with three to five frozen fire ant pupae and cleaned and watered the plaster as needed. Once the larvae had pupated, we counted the fluorescently labelled pupae. We measured mortality at the pupal stage because all genome sequencing to determine rates of loss of heterozygosity was performed using pupal offspring.

We harvested eggs for this experiment after allowing transgenic ants to lay eggs for 16 h. Thus, at the start of the experiment, the eggs ranged in age from 0 to 16 h after oviposition. Under our experimental conditions, embryonic development (until larval hatching) takes 12 days in *O. biroi*. Developmental mortality only becomes apparent to the experimenter after several days into development and should therefore be fully captured in this experiment.

One caveat might be if ants could immediately detect and remove (cannibalize) eggs that bear a loss of heterozygosity. This seems highly unlikely, however. First, loss of heterozygosity should only cause developmental phenotypes after gene expression from the offspring has begun. The maternal-to-zygotic transition occurs ~10% of the way through embryonic development in *D. melanogaster*, which in *O. biroi* would be >24 h after egg laying and almost certainly after the 16 h cutoff. Second, our behavioural observations suggest that egg cannibalization under these experimental conditions is absent or exceedingly rare. Taken together, it is therefore highly unlikely that we underestimate developmental mortality due to early embryonic mortality and egg removal in this experiment.

### Data visualization

Karyoplots were drawn using karyoploteR^63^, binomial probability mass functions were drawn using base R graphics^41^ and other plots were drawn using ggplot2 (ref. ^64^). Illustrations and final figures were made using Adobe Illustrator.

### Statistics

We constructed linear models using the lm() function in R v.4.0.5 (ref. ^41^). These were used to determine the slope and *y* intercept of the relationship between the normalized number of crossovers and phylogenetic distance among haploid males. We also used linear models as an internal control to confirm the validity of detecting crossovers in linked-read sequencing data by assessing whether the number of crossovers scaled positively with the number of meioses.

Only one of the 144 crossovers observed in our linked-read sequencing experiment was associated with a loss of heterozygosity. To determine whether these data differed significantly from the Mendelian expectation that two-thirds of inherited crossovers should be associated with losses of heterozygosity, we conducted a binomial test in GraphPad Prism.

To calculate the 95% CI of the proportion of offspring that bear losses of heterozygosity, we used the Wilson/Brown^65^ method in GraphPad Prism. This method is recommended for proportions very close to zero or one. We used standard methods to calculate all other 95% CIs.

A one-tailed, one-sample Wilcoxon test was used to determine if the fitness of the transgenic line was less than that of wild-type ants.

### Reporting summary

Further information on research design is available in the Nature Portfolio Reporting Summary linked to this article.

### Supplementary information

Reporting Summary

Supplementary Tables Supplementary Table 1: Transgenesis efficiency. Overall efficiency was calculated as the number of independent lines generated divided by the number of embryos injected. Transformation efficiency was calculated as the number of independent lines divided by the number of eclosed G0s. Table 2: Sample metadata. Metadata for all DNA whole-genome shotgun sequencing libraries included in this study. Table 3: Crossovers detected in comparisons of phased genomes. Each row reports one observed crossover, where the relative phasing of samples changed from SNP1 to SNP2. If loss of heterozygosity occurred in the vicinity of the crossover, the identity of the SNPs at which it occurred and the minimum and maximum tract lengths of this loss of heterozygosity event are given. LOH, loss of heterozygosity.

Supplementary Video *Z*-stack images of a DAPI-stained metaphase I nucleus taken at intervals of 0.17 µm. This oocyte was incompletely squashed and so the alignment of chiasmate bivalent chromosomes during metaphase can be clearly seen. Additional replicate oocytes are in Fig. 1 and Extended Data Fig. 1.

Supplementary Data 1 Haplotype blocks and loss-of-heterozygosity tracts produced by crossing over and gene conversion among individuals from the same colony, including events observed among haploid male genomes and inferred via loss of heterozygosity among diploid female genomes. For haplotype blocks among haploids, the values indicate phasing relative to the first individual—values of ‘0’ indicate that this individual has the same allele as the first individual for this haplotype, whereas values of ‘1’ indicate that this individual possesses the alternate allele. For losses of heterozygosity among diploids, values of ‘1’ mean that an individual possesses that loss-of-heterozygosity tract, whereas values of ‘0’ indicate that an individual is heterozygous for that tract. For crossovers inferred from loss-of-heterozygosity tracts, crossovers are counted at the beginning or end of a loss-of-heterozygosity tract if it was not also the first or last SNP on a contig.

Supplementary Data 2 Haplotype blocks and loss-of-heterozygosity tracts produced by crossing over and gene conversion among individuals from different colonies, including events observed among haploid male genomes and inferred via loss of heterozygosity among diploid female genomes. For haplotype blocks among haploids, the values indicate phasing relative to the first individual—values of ‘0’ indicate that this individual has the same allele as the first individual for this haplotype, whereas values of ‘1’ indicate that this individual possesses the alternate allele. For losses of heterozygosity among diploids, values of ‘1’ mean that an individual possesses that loss-of-heterozygosity tract, whereas values of ‘0’ indicate that an individual is heterozygous for that tract.

Supplementary Data 3 Loss-of-heterozygosity tracts produced by crossing over and gene conversion among individuals from different known pedigrees from linked-read sequencing experiments. Values of ‘1’ mean that an individual possesses that loss-of-heterozygosity tract, whereas values of ‘0’ indicate that an individual is heterozygous for that tract.

Supplementary Data 4 Crossovers detected in all pairwise comparisons between representatives from each linked-read sequencing pedigree. For each crossover, we label the two different haplotypes for the two focal SNPs as 0 and 1.

### Source data

Source Data Fig. 1 Statistical source data.

Source Data Fig. 2 Statistical source data.

Source Data Fig. 3 Statistical source data.

Source Data Fig. 4 Statistical source data.

## Figures and Tables

**Extended Data Table 1:**
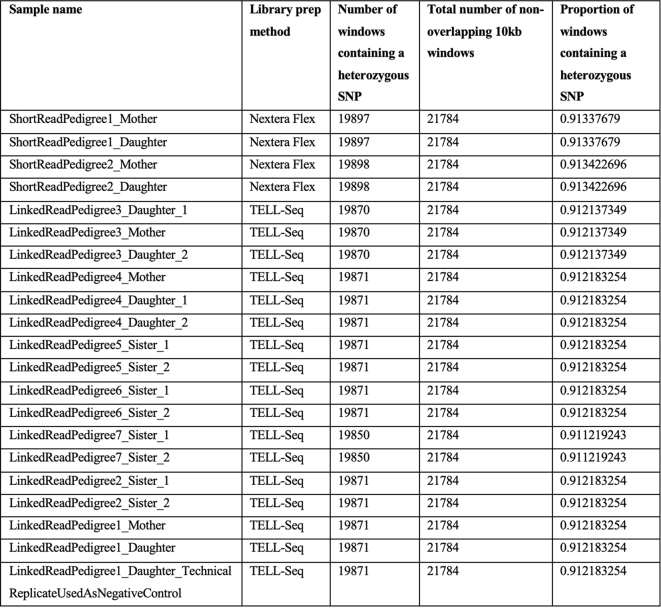
Genome-wide heterozygosity maintenance

Genome-wide heterozygosity data for all individuals from known pedigrees.

**Extended Data Table 2:**
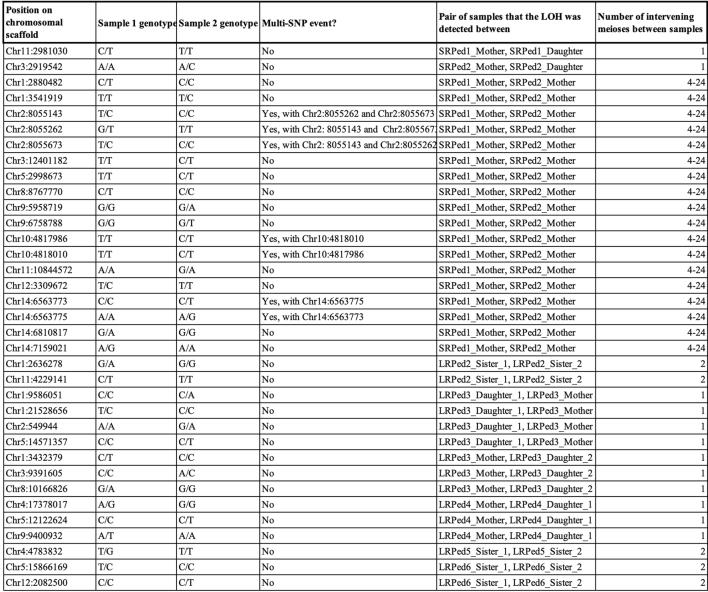
Changes of heterozygosity in known pedigrees

All SNPs that lost or gained heterozygosity in known-pedigree pairs. LOH: loss of heterozygosity.

## Data Availability

All DNA sequencing data are publicly available at the National Center for Biotechnology Information Sequence Read Archive under accession no. PRJNA947942. All other data are available in the article and Supplementary Information. Source data are provided with this paper.

## Code availability

All code is available on GitHub (https://github.com/kipdlacy/UnselfishMeioticDrive).
